# Evaluating clinical characteristics and neuroimaging indications of paediatric traumatic brain injury patients using Scandinavian paediatric traumatic brain injury guidelines in Southwest Finland

**DOI:** 10.1016/j.bas.2026.105969

**Published:** 2026-02-12

**Authors:** Nina Erkinjuntti, Jussi P. Posti, Tuire Lähdesmäki

**Affiliations:** a Paediatric Neurology Department, Oulu University Hospital, Finland; b Neurocenter, Department of Neurosurgery and Turku Brain Injury Centre, Turku University Hospital, Finland; c Paediatric Neurology, Department of Paediatrics and Adolescent Medicine, Turku University Hospital, Finland; d Division of Clinical Neurosciences, University of Turku, Turku, Finland; e Division of Paediatrics and Adolescent Medicine, University of Turku, Turku, Finland

**Keywords:** Paediatric traumatic brain injury, Neuroimaging, Scandinavian guidelines

## Abstract

**Introduction:**

Neuroimaging of paediatric head injuries has increased during the past decades.

**Research question:**

To describe clinical features and neuroimaging outcomes of the retrospective cohort, and to assess the alignment of neuroimaging decisions for paediatric head injuries with the Scandinavian guidelines for the management of mild and moderate head trauma in children (SCN16 guideline) in a tertiary care setting.

**Materials and methods:**

Patients <16 years with head injury and neuroimaging were identified through diagnostic registry search at Turku University Hospital (2010–2016), Finland. Medical records were reviewed for data collection. The SCN subgroup included patients assessed within 24 h and with a Glasgow Coma Scale (GCS) of 13–15.

**Results:**

Of the 637 patients (58% male, mean age 9.3 years), traumatic intracranial pathologies were found in 9% (n = 55) on CT and 13% (n = 85) on MRI; 3% (n = 16) underwent neurosurgery. Most skull fractures (82%, n = 31) co-occurred with intracranial findings. Between 2010 and 2016, CT use increased from 37 to 70 and MRI from 20 to 93, while traumatic abnormalities remained stable or decreased. 512 patients were included in the SCN subgroup (59% male, mean age 9.4). Acute neuroimaging was performed in 75%; 91% fulfilled the SCN16 neuroimaging criteria. Two (0.4%) of 163 discharged patients required neurosurgery, and 14 (3%) had traumatic pathology on later neuroimaging.

**Discussion and conclusions:**

In this cohort, neuroimaging indications during 2010–2016 largely aligned with the SCN16 guidelines. Clinical features, neuroimaging utilization, and neurosurgical intervention rates were generally consistent with previous literature.

## Highlights

• Neuroimaging guidelines ensure proper patient selection and appropriate admission.
• Most skull fractures presented with traumatic intracranial findings (84%).
• MRI use for selected head trauma patients may be a consideration in certain cases.

## 1 Introduction

Traumatic brain injury (TBI) is a significant health concern reaching beyond demographic and societal boundaries for children and adolescents worldwide (Dewan et al., 2016; Majdan et al., 2022). Traumatic intracranial pathology varies widely in TBI depending on the severity of the trauma. The initial symptoms of the brain injury may resolve quickly in the milder forms of TBI. Still, some patients suffer from prolonged symptoms that can induce functional disabilities of various severity (Lumba-Brown et al., 2018; Lloyd et al., 2015; Yuh et al., 2013). The worldwide incidence of paediatric TBI ranges widely; most reported rates are between 47 and 280/100,000, with a male predominance of 1.8:1 on average (Dewan et al., 2016; Majdan et al., 2022). The estimated incidence of paediatric TBI has increased in Finland over the years, being 332/100,000 between 1998 and 2018, with a male predominance of 1.3:1 (Kuitunen et al., 2023). The reported cumulative incidence of hospitalized TBI has been estimated at 345/100,000 in Europe and 99–333/100,000 in Finland among youth and adolescents (Majdan et al., 2022; Kuitunen et al., 2023; Wilson M et al., 2017). Known risk factors for TBI, in addition to male gender, are age of 15–19 years, previous TBI, contact sports, and the ignorance of using safety equipment such as helmets or seatbelts (Cassidy et al.; McKinlay et al., 2010).

Mechanisms of injury range across populations and age groups. Motor vehicle collisions and falls are the significant mechanisms of injury globally, followed by abuse and other forms of non-accidental trauma (Dewan et al., 2016; Majdan et al., 2016). In Finland, falls are the primary mechanism of injury in all age groups, and traffic accidents, especially in youth aged 10–19 (Koskinen and Alaranta, 2008; Posti et al., 2023).

The vast majority (80–90%) of brain injuries are classified as mild or minor (Kuitunen et al., 2023; Astrand et al., 2016). The TBI severity classification is usually based on information about Glascow Coma Scale (GCS), post-traumatic amnesia (PTA), loss of consciousness (LOC), and traumatic intracranial findings in neuroimaging (Aivovammat. Suomalaisen Lääkäriseuran Duodecimin, 2023). Mortality and a need for neurosurgical interventions are rare in the group of mild TBI (mTBI), but 4–6% have traumatic intracranial findings on the head computed tomography (CT) scan (Astrand et al., 2016; Kuppermann et al., 2009).

Owing to the high incidence of paediatric TBI, the fact that most TBIs are classified as minor, and the increasing number of CT scans done for children (Koskinen and Alaranta, 2008; Astrand et al., 2016; Qvist et al., 2019), there is a need for clinical practice guidelines for the acute evaluation of head injury patients. Several algorithms have been developed to aid decision-making in neuroimaging and hospital admission following head injury (Astrand et al., 2016; Kuppermann et al., 2009; Osmond et al., 2018; Undén et al., 2018). In addition to recognizing TBI and the patients needing neurosurgery intervention, these decision rules aim to reduce head CT usage as CT imaging exposes children to ionizing radiation, and increases healthcare costs (Lesyk et al., 2024). In the validation study of one of these decision rules, the Scandinavian guidelines for initial management of minor or moderate head trauma in children (SCN16 guidelines), sensitivities for detecting the need for neurosurgery, clinically important TBI, and brain injury on CT have been reported to be 100%, 98%, and 95% (Undén et al., 2018). Additionally, a recent validation study of the SCN16 guideline demonstrated an adequate diagnostic performance in a real-world cohort (Wickbom et al., 2025).

Due to increased hospital admissions and neuroimaging of paediatric head injury patients at our tertiary care centre, we sought to study the clinical characteristics and neuroimaging indications of these patients in greater detail. In this paper, we present the clinical features and neuroimaging findings of the paediatric TBI population from 2010 to 2016. To assess real-life clinical decision-making in more detail, this paper also compares real-life acute neuroimaging indications at a single centre (2010-2016) with the 2016 SCN guideline (Wickbom et al., 2025).

## 2 Materials and methods

### 2.1 Participants

The cases were identified through a diagnosis-based register search performed by the Turku Clinical Research Centre (Turku CRC), covering visits and neuroimaging examinations 2010–2016 at the Turku University Hospital (TUH) Department of Paediatrics and Adolescent Medicine and Emergency Departments. We included patients who met the following criteria: 1) age under 16 years, 2) diagnosis identified by ICD-10 codes corresponding to the following TBI categories: S06.∗ (intracranial injuries), T90 (sequelae of injuries of the head), F07 (personality and behavioral disorders due to brain disease, damage, and dysfunction), and F04 (organic amnesic syndrome, not induced by alcohol and other psychoactive substances), and 3) had head CT and/or MRI imaging due to head injury.

The patients were excluded 1) if they had features of severe TBI, or 2) if the initial evaluation of the head injury lacked sufficient clinical information.

Information about age, sex, injury mechanisms, neurosurgical interventions, neuroimaging modalities, and findings is reported. The patient's sex was determined based on the personal identity code recorded in Finland's Population Information System at the time of data collection. As a result, this information may not align with the patient's gender identity.

**Chart 1:**
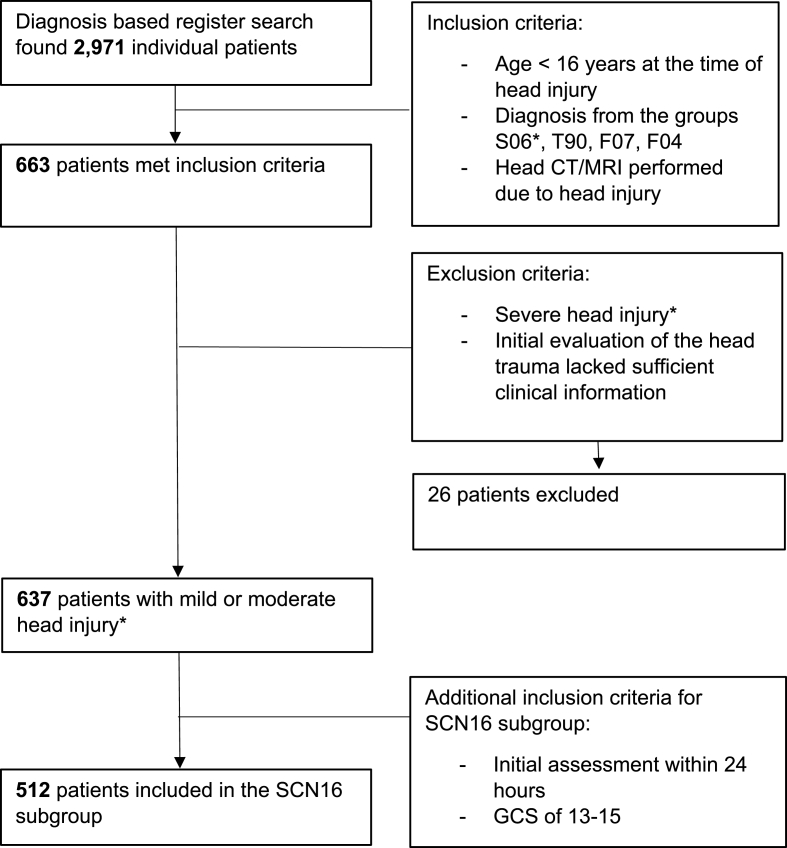
Inclusion process of the study ∗Severity of injury was defined as follows: Mild TBI: GCS of 13-15, LOC <30 min, PTA <24 h and no traumatic intracranial abnormalities in neuroimaging. Moderate TBI: GCS of 9-12, LOC >30 min but <24 h, and PTA >24 h but <7 days, and traumatic intracranial pathologies. Severe TBI: GCS of 8 or less, LOC >24 h, PTA >7 days, and traumatic intracranial pathologies on neuroimaging.

The information needed for clinical decision-making (injury-related symptoms) was assessed to evaluate the neuroimaging indications. Further details about the inclusion process are shown in Chart 1.

To compare neuroimaging indications with SCN16 guidelines, a “SCN subgroup” was created based on SCN16 criteria, but limited by the availability of exact GCS scores and LOC duration. Additional inclusion criteria for this group were 1) initial assessment within 24 h, and 2) GCS of 13–15. In this paper, acute neuroimaging refers to head neuroimaging performed within 24 h after head injury. Patients were classified into four severity groups (“minimal”, “mild-low risk”, “mild-moderate risk”, and “mild-high risk”) based on injury-related symptoms as in SCN16 guidelines (Astrand et al., 2016), with each individual assigned to the group corresponding to their highest-risk symptom, even if they also presented with symptoms from lower-risk categories. The “moderate risk” group was not formed, as in this study, the GCS score was collected as a categorical variable with a range other (GCS of 13-15) than in SCN16 guidelines (GCS of 9-13 and 14-15).

The neuroimaging indications were evaluated according to the SCN16 criteria; the guidelines recommend CT imaging as follows: 1) patients classified as “moderate” or “mild high-risk”, 2) “mild medium-risk” patients as an alternative to observation for ≥12 h, and 3) “mild low-risk” patients with multiple risk factors. Neuroimaging is not recommended for patients with “minimal risk”. SCN16 guidelines recommend hospital admission for patients with 1) a suspicion of non-accidental injury, 2) high-energy trauma mechanisms (road traffic accidents, falls from >3 m height), and 3) patients under 1 year of age, regardless of symptoms. As stated in the SCN16 guidelines, high-speed traffic accidents and falls >3 m of height are typically managed according to trauma protocol and whole-body trauma CT. This is also the practice at our centre; therefore, these patients are considered to meet the neuroimaging criteria but are reported separately.

GCS was assessed based on clinical information in patients’ medical records, as it was rarely marked numerically. Prehospital and worst GCS data were collected, but only the worst GCS was reported, consistent with previous studies from our centre (Posti et al., 2016, 2019). Data on PTA in different time categories were gathered, but it was grouped into ”no PTA ”and ”PTA over 10 min” for analysis. If PTA was considered under 10 min, it was classified as ”no PTA.” The information about the LOC was categorized into groups of “no LOC” and “LOC <30 min”. In the case of uncertain information about LOC or PTA, it was marked as “unknown”. Data on other symptoms related to head injury were also collected. Focal neurological deficit was defined as any deficit or pathological finding in the clinical neurological examination (e.g., paresis of the extremities, cranial nerve affection, anisocoria, ataxia, or aphasia).

### 2.2 Neuroimaging

Information about head neuroimaging modalities and traumatic intracranial pathologies was collected from the medical records. During the study period, neuroimaging examinations were performed in accordance with the local protocol for acute head injuries. There were no national recommendations or guidelines for the initial management of head injuries for youth and adolescents. In an acute setting, either a head CT or an MRI was performed. MRIs were performed at the TUH or Terveystalo, a private healthcare service provider in Turku, Finland, while all the head CTs were performed at the TUH. Head CTs and MRIs were primarily assessed by a neuroradiology specialist and, in some cases, a radiology specialist. Common Data Elements (CDE) were utilized to plan data collection and classification of the neuroimaging findings (Duhaime et al., 2010, 2012). In this study, more than three microhemorrhages detected on susceptibility-weighted imaging (SWI) or other hemosiderin-sensitive MRI sequences were considered indicative of diffuse axonal injury (DAI).

### 2.3 Data collection and analysis

Information was gathered from medical records by N.E. In cases that were unclear, J.P.*P. and* T.L. were consulted. Data was collected using Microsoft Excel (version 2407, Microsoft Corporation, Redmond, Washington, United States of America).

The Chi-square test for independence was used to test differences among the categorical variables across SCN severity groups. The cases were excluded from the analysis if any individual case was missing data required for the specific analysis. A p-value of <0.05 was considered statistically significant. Analyses were performed using SPSS, version 27.0 (IBM SPSS Statistics; IBM Corp, Armonk, NY).

## 3 Results

### 3.1 The whole study population

A total of 2971 patients were screened after a diagnosis-based register search. 663 patients met the initial inclusion criteria. After exclusion the study population consisted of 637 patients with minor or moderate head injury. Excluded patients (n = 26) had severe head injury (n = 16) or lacked information about head injury evaluation in the medical records (n = 10). As shown in Table 1, the mean age of patients was 9.2 years (range 0.02-15), 48% (n = 304) of the head injuries occurred in the age group of 11-15 years. 58% were male (n = 371). The most reported head injury-associated risk factors were LOC <30 min (24%, n = 155), focal neurological deficits (27%, n = 171), and severe or progressive headache (25%, n = 162). The most common cause of injury was an incidental fall (46%, n = 290). 18% (n = 115) of head injuries were sport-related. Supplementary Table 1 presents the causes of injury among age groups and sexes.

**Table 1:** Demographics, clinical characteristics, and outcome measures of the study population.

	SCN subgroup^a^	All
n	512	637
**Age and sex**		
Mean	9.39	9.25
Median (range)	10 (0.02-14.98)	10 (0.02-14.98)
<1 year	19 (3.7)	28 (4.4)
1-5 years	109 (21.3)	138 (21.7)
6-10 years	136 (26.6)	167 (26.2)
11-15 years	248 (48.4)	304 (47.7)
Male	303 (59.2)	371 (58.2)
Female	209 (40.8)	266 (41.8)
**Cause of injury**		
Incidental fall	233 (45.5)	290 (45.5)
Other non-intentional injury	172 (33.6)	227 (35.6)
Violence/assault	12 (2.3)	17 (2.7)
Traffic	95 (18.6)	103 (16.2)
Sports-related injuries	94 (18.4)	115 (18.1)
**Clinical characteristics**		
GCS		
GCS 13-15	512 (100.0)	619 (97.2)
GCS 9-12		10 (1.6)
GCS 3-8		3 (0.5)
Unknown		5 (0.8)
LOC		
No LOC	306 (59.8)	387 (60.8)
LOC <30 min	128 (25.0)	155 (24.3)
Unknown	78 (15.2)	95 (14.9)
PTA		
No PTA	176 (34.4)	220 (34.5)
PTA >10 min	119 (23.2)	132 (20.7)
Unknown	217 (42.4)	285 (44.7)
**Other risk factors**		
Focal neurological deficit	122 (23.8)	171 (26.8)
Post-traumatic seizures	33 (6.4)	38 (6.0)
Clinical signs of skull base fracture or depressed skull fracture	8 (1.6)	11 (1.7)
Anticoagulation or coagulation disorder	1 (0.2)	1 (0.2)
Severe/progressive headache	121 (23.6)	162 (25.4)
Abnormal behavior according to the guardian	20 (3.9)	24 (3.8)
Vomiting ≥2 times	111 (21.7)	132 (20.7)
Shunt	0	1 (0.2)
If age <2: large, temporal or parietal scalp hematoma	9 (1.8)	14 (2.2)
Irritability	10 (2.0)	13 (2.0)
Multiple risk factors	160 (31.3)	209 (32.8)
**Outcome measures**		
Acute head CT	317 (61.9)	334 (52.4)
All CTs	356 (69.5)	411 (64.5)
TIP on acute CT	30 (5.9)	40 (6.3)
TIP on CT performed later	9 (1.8)	15 (2.4)
Acute head MRI	66 (12.9)	67 (10.2)
All MRIs	292 (57.0)	395 (62.0)
TIP on acute MRI	10 (2.0)	11 (1.7)
TIP on MRI performed later	55 (10.7)	74 (11.6)
Discharged	163 (31.8)	221 (34.7)
Admitted to the hospital	349 (68.2)	416 (65.3)
Extracerebral injuries	352 (68.8)	425 (66.7)
Neurosurgical intervention	12 (2.3)	16 (2.5)

Data refers to the number of patients (% of the group).

GCS = Glasgow Coma Scale, LOC = Loss of consciousness, PTA = Post-traumatic amnesia, CT = computed tomography, MRI = magnetic resonance imaging, TIP = traumatic intracranial pathology.

Acute head neuroimaging = neuroimaging within 24 h after head injury.

Eighteen patients with a GCS of less than 13 or an unknown GCS underwent initial neuroimaging (17 had a CT scan, 1 had an MRI) and were excluded from the subgroup analysis.

a SCN subgroup of patients with healthcare evaluation <24 h of trauma and the worst GCS of 13-15.

During the study period, 806 neuroimaging examinations were performed on 637 patients. 51% (n = 411) of these were head CTs. MRI was performed in 35% (n = 226) as the only neuroimaging modality. Both head CT and MRI scans were conducted on 27% (n = 169) of the study population. 72% (n = 286) of the MRIs were performed with a 3 T MRI device. 84% (n = 333) of the MRIs were performed with a trauma protocol including T1-and T2-weighted sequences, FLAIR, susceptibility-weighted (SWI), and diffusion-weighted (DWI) sequences.

Of the whole study population, 9% (n = 55) had traumatic intracranial pathologies on CT and 13% (n = 85) on MRI. The most common findings on CT included skull fractures (9%, n = 38), contusions and extra-axial hematomas, each observed in 6% (n = 23). Of all the patients with skull fractures (n = 38), seven (18%) had isolated skull fractures. MRI revealed DAI and contusions in 11% (n = 44) of cases each, and extra-axial hematomas were observed in 9% (n = 37). Of the patients with both CT and MRI (n = 169) (Table 3), 50 (30%) had traumatic intracranial pathology on CT, 9% (n = 15) had normal CT but traumatic intracranial pathology on MRI. Of these patients, 14/15 had DAI, and 1/15 had contusions. Two patients had an extra-axial hematoma in addition to contusion or DAI.

**Table 2:** Neuroimaging modalities, neuroimaging findings and neurosurgical interventions.

Neuroimaging modalities, neuroimaging findings and neurosurgical interventions			
	Subgroup		All
	Acute CT/MRI	Later CT/MRI	
Head CT	n = 317	n = 39	n = 411
**Imaging delay**			
Mean (range)	0	2.31	0.46
Median	0	1 (1-19)	0 (0-19)
Skull fractures	17 (5.4)	8 (20.5)	38 (9.2)
Contusions	14 (4.4)	3 (7.7)	23 (5.6)
Extra-axial hematomas	13 (4.1)	3 (7.7)	23 (5.6)
Other	3 (0.9)	2 (5.1)	5 (1.2)
**Head MRI**	n = 66	n = 226	n = 395
**Imaging delay**			
Mean (range)	0	24.0	17.8
Median	0	11.0 (1-548)	5.0 (0-549)
Imaging scanner strenght			
3 T	63 (95.5)	153 (67.7)	286 (72.4)
1,5 T	3 (4.5)	73 (32.3)	109 (27.6)
MRI performed due to CT finding	2 (3.0)	33 (14.6)	50 (12.7)
Traumaprotocol	61 (92.4)	182 (80.5)	333 (84.3)
Skull fractures	1 (1.5)	12 (5.3)	19 (4.8)
Contusions	7 (10.6)	28 (12.4)	44 (11.1)
DAI	3 (4.5)	32 (14.2)	44 (11.1)
Extra-axial hematomas	4 (6.1)	24 (10.6)	37 (9.4)
Other	1 (1.5)	4 (1.8)	5 (1.3)
**Neurosurgical interventions**	n = 512		n = 637
ICP-measurement	2 (0.4)		4 (0.6)
Craniotomy and hematoma evacuation	10 (2.0)		12 (1.9)
Skull reconstruction	4 (0.8)		4 (0.6)
Trepanation	0		1 (0.2)

Data is number of findings/interventions (% of the group). Some patients may have several findings/interventions.

Other findings included intraventricular hemorrhage, brain edema, supratentorial midline shift, cisternal compression.

**Table 3:** Neuroimaging of patients with both CT and MRI (n = 169).

	CT	MRI	CT -/MRI +	CT + /MRI -
**All findings (no. Patients, % of the subpopulation n = 169)**	50 (29.6)	56 (33.1)	15 (8.9)	9 (5.3)
**Traumatic intracranial findings (no. findings)**				
Skull fracture^c^	34 (68.0)	14 (25.0)		6 (66.7)
Contusion	21 (42.0)	27 (48.2)	1 (6.7)	
DAI		33 (58.9)	14 (93.3)	
Extra axial hematoma	21 (42.0)	26 (46.4)	2 (13.3)	2 (22.2)^a^
Other^b^	5 (10.0)	3 (5.4)		2 (22.2)

Data is the number of patients/findings, (% of the group).

CT - or MRI - = no traumatic intracranial findings, CT + or MRI + = traumatic intracranial findings.

DAI = diffuse axonal injury.

a One patient with an extra-axial hematoma had neurosurgical intervention, and two had an MRI after the hematoma had resorbed.

b Other findings included intraventricular hemorrhage, brain edema, supratentorial midline shift, and cisternal compression.

c Seven patients had isolated skull fractures with no traumatic intracranial findings (6/7 of the isolated fractures detected on CT, and one on MRI performed as a primary neuroimaging modality).

Of the whole study population, 3% (n = 16) required neurosurgical intervention, and 65% (n = 416) were admitted to the hospital. The mean age of patients admitted to the hospital was 8.5 years, and 10.6 years for those not admitted. 67% (n = 425) had extracerebral injuries in addition to head injury. Information about patient demographics, clinical characteristics, and neuroimaging modalities and findings is presented in Table 1, Table 2, Table 3. Supplementary Table 2 presents the distribution of extra-cerebral injuries by sex, age groups, and causes of injury.

**Fig. 1:**
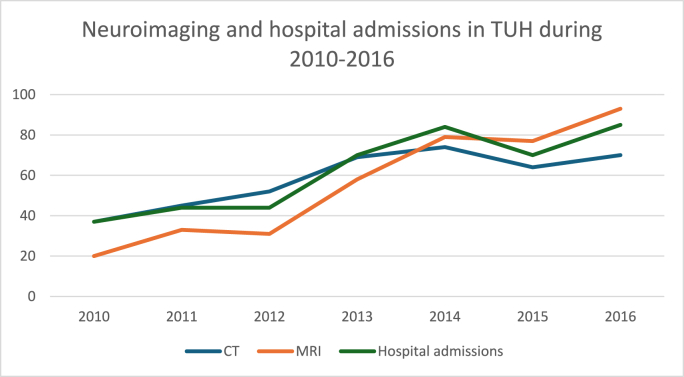
Trends of neuroimaging and hospital admissions in Turku University Hospital (TUH) during the study period 2010-2016.

**Fig. 2:**
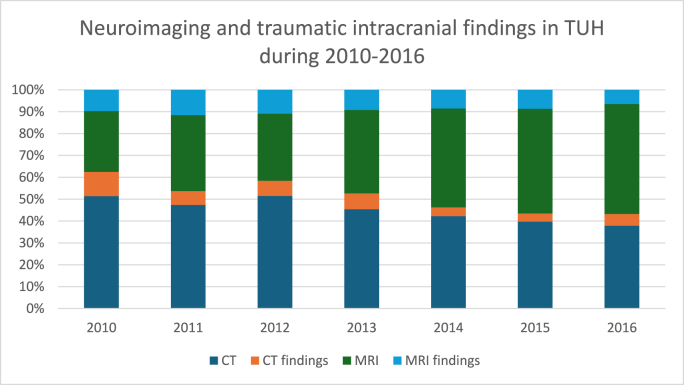
Neuroimaging and traumatic intracranial findings in TUH.

**Fig. 3:**
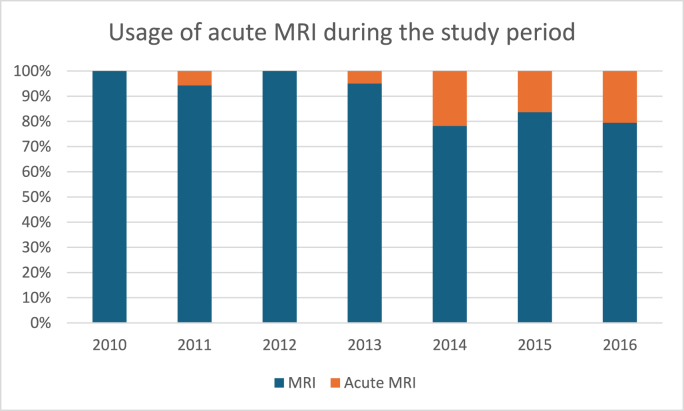
Usage of acute MRI imaging during 2010-2016 in TUH.

The number of neuroimaging examinations, including head CTs and MRIs, increased during the study period, whereas the percentage of traumatic intracranial findings decreased or remained stable across both modalities. MRI was used more frequently as an acute neuroimaging modality towards the end of the study period: during 2014-2016, 20-28% of all MRIs were performed in an acute setting, whereas at the beginning of the study period, it was used in 0-6% of patients. Trends in overall neuroimaging, percentages of neuroimaging, traumatic intracranial pathologies, and hospital admissions are presented in Fig. 1, Fig. 2, Fig. 3.

### 3.2 SCN subgroup

Among patients with neuroimaging indications compared with SCN16 guidelines (n = 512), 59% (n = 303) were male, with a mean age of 9.39 years. The most common cause of injury was incidental falls (n = 233, 46%).

Among patients who underwent acute head neuroimaging (<24 h) (n = 383), the majority (91%; n = 350) met the SCN16 neuroimaging criteria. When traffic accidents are classified as high-energy injury mechanisms, the proportion of patients meeting the SCN16 criteria increases to 95% (n = 364). As shown in Table 1, most patients with acute neuroimaging (n = 383, 75%) underwent acute CT (n = 317), while the rest (n = 66) had acute MRI. Table 4 presents the distribution of patients when classified according to SCN16 criteria.

**Table 4:** Cohort distribution (SCN subgroup) when classified according to SCN16 guidelines, acute neuroimaging, traumatic intracranial findings, hospital admissions, and neurosurgical interventions.

	Children <16 years after minor or moderate head trauma within 24 h of trauma (n = 544)			
	GCS 13-15 (n = 512)			
	AND			
**Riskfactors**	**Mild-high risk (n = 158)**	**Mild-moderate risk (n = 79)**	**Mild-low risk (n = 238)**	**Minimal risk (n = 37)**
	Focal neurological deficit, or post-traumatic seizure, or clinical signs of skull base fracture, or depressed skull fracture	LOC <30 min, or anticoagulation or coagulation disorder	PTA, or severe/progressive headache, or abnormal behaviour according to guardian, or vomiting ≥2, or suspected LOC, or shunt, or if age <2 years: large temporal or parietal scalp hematoma, or irritability	No risk factors, no PTA, no LOC

**aCT**	85 (53.8)	56 (70.9)	148 (62.2)	28 (75.7)
**aCT TIP**	10 (6.3)	8 (10.1)	12 (5.0)	0
**aMRI**	28 (17.7)	2 (2.5)	31 (13.0)	5 (13.5)
**aMRI TIP**	3 (1.9)	0	7 (2.9)	0
**Hospital admission**	107 (67.7)	56 (70.9)	165 (69.3)	21 (56.8)
**Neurosurgical intervention**	6 (3.8)	1 (1.3)	5 (2.1)	0

	Discharged (n = 51)^a^	Discharged (n = 23)^a^	Discharged (n = 73)^a^	Discharged (n = 16)^a^
	Neurosurgical interventions (n = 0)	Neurosurgical interventions (n = 0)	Neurosurgical interventions (n = 2)^b^	Neurosurgical interventions (n = 0)
	CT TIP (n = 2)	CT TIP (n = 0)	CT TIP (n = 3)	CT TIP (n = 0)
	MRI TIP (n = 3)	MRI TIP (n = 2)	MRI TIP (n = 6)	MRI TIP (n = 0)

GCS = Glasgow Coma Scale, aCT = acute head computed tomography, aMRI = acute magnetic resonance imaging, TIP = traumatic intracranial pathology.

High-velocity injury mechanisms (such as traffic accidents) are the probable cause of neuroimaging in a group with “minimal risk” in 18 patients (48.6%).

17/19 of patients <1 year of age were admitted to the hospital.

Traumatic intracranial findings on discharged patients included fractures (Lloyd et al., 2015), contusions (Cassidy et al.), DAI (Yuh et al., 2013), and extra-axial hematomas (Lumba-Brown et al., 2018).

a Discharged at first evaluation but re-evaluated later.

b Both patients had extra-axial hematomas needing neurosurgical intervention.

The mean and median delays for later (>24 h) neuroimaging were 2.31 and 1.0 days (range, 1-19 days) for head CT, and 24.0 and 11.0 days (range 1-548 days) for head MRI, respectively. Traumatic intracranial pathology was identified on acute CT in 12 cases (5%) in the “mild-low risk” group, 8 cases (10%) in the “mild-moderate risk” group, and 10 cases (6%) in the “mild-high risk” group. On acute MRI, traumatic intracranial pathology was detected in 7 cases (3%) in the “mild-low risk” and 3 cases (2%) in the “mild-high risk” groups. Neuroimaging modalities and findings across different groups are presented in Table 2, Table 4, and Supplementary Table 3.

Of the patients with “minimal risk”, 57% (n = 21) were admitted to the hospital, while hospital admissions occurred in 69% (n = 165), 71% (n = 56), and 68% (n = 107) in groups with “mild-low risk”, “mild-moderate risk”, and “mild-high risk”. Of the discharged patients (n = 163, 32%), two were re-evaluated due to symptom worsening, diagnosed with extra-axial hematomas, and needed neurosurgical intervention. In addition, 16 discharged patients had traumatic intracranial pathology on later neuroimaging. These traumatic intracranial pathologies included fractures, contusions, DAI, and extra-axial hematomas.

Neurosurgical interventions were performed on 12 patients (2%). The most common interventions were craniotomy and hematoma evacuation, performed on 10 patients (2%).

The SCN subgroups showed statistically significant differences regarding sex (p = 0.013), age (p=<0.001), and causes of injury (p=<0.001). There were also statistically significant differences between the groups regarding extra-cerebral injuries (p = 0.032). This data is presented in Supplementary Tables 3 and 4

## 4 Discussion

This study examined the clinical characteristics of paediatric patients with head injury who underwent neuroimaging in a paediatric tertiary care centre in Southwest Finland between 2010 and 2016. It also examined the neuroimaging protocol used at our centre between 2010 and 2016 and compared it with the indications of the SNC16 guideline, which later became a European standard.

The number of neuroimaging examinations, including head CTs and MRIs, increased during the study period, while the number of traumatic intracranial pathologies remained unchanged or decreased. Head MRI was used more frequently as an acute neuroimaging modality towards the end of the study period. Of the whole study population, 9% (n = 55) had traumatic intracranial pathology on CT and 13% (n = 85) on MRI. Of all the patients with skull fractures (n = 38), seven patients (18%) had isolated skull fractures. Of the patients who underwent acute neuroimaging (n = 383), 91% (n = 350) would have met the SCN16 neuroimaging criteria if those guidelines had been applied during the study period, rising to 95% (n = 364) when traffic accidents were classified as high-energy trauma. Two patients (0.4%) with symptoms related to “mild-low risk” were discharged but needed re-evaluation and neurosurgical intervention. In addition, 16 discharged patients (3%) of the SCN subgroup with “mild-low risk”, “mild-moderate risk”, and “mild-high risk” had traumatic intracranial pathology in later neuroimaging.

The demographics of the study population were comparable to those in most previous epidemiological studies (Dewan et al., 2016; Majdan et al., 2022; Kuitunen et al., 2023; Schneier et al., 2006; Winqvist et al., 2007). Neuroimaging increased over the study period, largely due to its use in milder head injury cases. This same observation was also made in another Finnish study (Kuitunen et al., 2023). Several reasons might explain this. Firstly, greater awareness of possible sequelae related to head injuries among physicians and families has resulted in larger numbers of TBI diagnoses being made for patients with milder symptoms, and families seeking health care evaluation. Secondly, during the study period, some structural changes were made to the healthcare system in Southwestern Finland, which may have improved the availability of neuroimaging. Finally, the use of clinical practice guidelines varies, and their implementation remains unclear.

The number of head injury patients with positive neuroimaging ranges widely between studies (Dewan et al., 2016). We report trauma-related findings on 9% of patients with head CTs and 13% with head MRIs, which is in line with previous reports (Dewan et al., 2016). Of the whole study population, 169 (27%) had both head CT and MRI. As knowledge of post-injury sequelae has grown, the importance of accurate neuroimaging for diagnosing all intracranial injuries has become increasingly evident. In our centre, all the paediatric patients with traumatic intracranial pathologies on CT also undergo MRI to further evaluate the extent of the traumatic intracranial pathologies. In this subpopulation, 50 (30%) patients had traumatic intracranial pathologies in CT scans. The reasons for repeated MRI in the remaining subpopulation may be related to injury-related or post-injury symptoms. The proportion of patients with normal CT but traumatic intracranial pathology on MRI (9%, n = 15) is consistent with previous paediatric studies (Roguski et al., 2015; Buttram et al., 2015). Differences in traumatic intracranial pathologies across neuroimaging modalities may be partly explained by the timing of imaging. In adult studies, a higher prevalence of MRI findings has been reported in patients with normal CT (Yue et al., 2019). The differences observed in adult studies may be attributable to variations in patient selection across neuroimaging modalities. Repetitive neuroimaging could be avoided by using MRI as the only neuroimaging modality in acute settings for selected patients, especially adolescents who do not require sedation during MRI.

In the validation study of SCN guidelines, only four patients out of 19,007 with clinically important TBI were missed, and none needed specific intervention (Undén et al., 2018). Additionally, a recent study evaluating the diagnostic performance of the SCN16 guidelines in a real-world cohort found results supporting their validity (Wickbom et al., 2025). In this study, the neuroimaging indications applied at our paediatric tertiary care centre from 2010 to 2016 mainly aligned with those suggested in the SCN16 guidelines. However, in our study, two patients (0.4%) requiring neurosurgical intervention were missed at initial evaluation, and there were also 16 discharged patients (3%) with traumatic intracranial pathology revealed in later neuroimaging. Therefore, our findings differed from those reported in a recent prospective real-world SCN16 validation study by Wickbom et al. (2025). Wickbom et al. reported that neurosurgical intervention was required in 0.07%, while clinically significant findings on head CT were observed in 0.9%. Also, no children with discharge recommendation needed neurosurgery, or had substantial trauma-related findings on head CT. Comparing these studies is challenging because of differences in inclusion criteria; we included only patients who underwent neuroimaging. Additionally, there are variations in patient age and GCS scores. Probable explanations for missing the patients needing neurosurgical interventions in our cohort include relatively mild symptom presentation and the short interval between head injury and the initial healthcare visit. Regarding the patients with traumatic intracranial pathologies in later CT imaging, the median delay for imaging was only one day; this may be considered a reasonable delay when evaluating head injuries not requiring neurosurgical intervention. Also, the median delay of 11 days for MRI following head injury falls within the recommended imaging timeframe according to Finnish Current Care Guidelines for TBI in adults (Aivovammat. Suomalaisen Lääkäriseuran Duodecimin, 2023). However, missing the children with neurosurgery requiring head injury highlights the importance of clinical practice guidelines. Children represent a special patient group with unique injury mechanisms that are often unwitnessed, appear minor, and are susceptible to clinical misjudgement.

Statistically significant differences between SCN groups were found regarding sex, age, causes of injury, and extra-cerebral traumas. No prior literature exists comparing SCN16 risk groups by patient demographics. Age and sex differences may reflect more severe injury mechanisms in males and adolescents, particularly high-velocity traffic accidents. Also, symptom recognition may be better in older children. Traffic accidents were a more common cause of injury in the “minimal risk” group than in other groups, and this may be related to imaging practices of high-velocity traumas. Differences in injury causes could explain discrepancies in extra-cerebral injuries between the groups.

Most patients with acute neuroimaging had a head CT (85%). MRI, as a primary neuroimaging method, increased towards the end of the study period, most likely due to better availability. Also, as described above, older age increased the likelihood of initial neuroimaging, while younger patients were more often hospitalized and may have received an MRI over a CT to avoid radiation. The on-duty physician or neuroradiologist selected the neuroimaging modality based on the clinical information and the MRI availability. It is also possible that the likelihood of using CT was higher late in the evening and at night due to issues related to the organization of emergency department services; however, this information was not collected during the data collection process. The usage of MRI in an acute setting has been evaluated in several study settings and paediatric populations, and its increased usage has been recognized (Marin et al., 2020; Cohrs et al., 2018). SWI has shown better sensitivity in detecting neuropathology than CT or MRI alone (Beauchamp et al., 2011; Roguski et al., 2015; Sarioglu et al., 2018; Provenzale, 2007). Rapid MRI, also known as QuickBrain MRI, has been evaluated in paediatric patients with TBI and has demonstrated good sensitivity and specificity in identifying clinically significant TBI (Sheridan et al., 2017; Cohen et al., 2015; Young et al., 2016). MRI was more precise in our study in recognizing intraparenchymal lesions such as contusions and DAI, as expected based on previous pediatric studies (Beauchamp et al., 2011; Roguski et al., 2015; Buttram et al., 2015). The need for neuroimaging after a head injury is twofold: firstly, recognizing the patients needing neurosurgical interventions or hospital admission is crucial, as discussed above. Head CT and MRI, as well as rapid MRI sequences, have demonstrated good sensitivity for this purpose. Secondly, neuroimaging may help predict functional outcomes after TBI and inform clinical decisions, particularly in mTBI. However, MRI, including SWI sequences and especially other advanced neuroimaging techniques, such as diffusion tensor imaging (DTI), and functional MRI (fMRI) is required for this purpose (Sheldrake et al., 2023; Yuh et al., 2014; Königs et al., 2018; Janas et al., 2022; Puig et al., 2020).

Guideline usage is one of the ways to reduce CT usage and hospitalizations in paediatric TBI without compromising safety. In our study, neuroimaging and hospitalizations increased during the study period despite the good alignment of the SCN16 guidelines assessed retrospectively. In a recent review using the Pediatric Emergency Care Applied Research Network (PECARN) head injury decision rule, the use of head CT imaging was reduced by a median of 27% (Lesyk et al., 2024). Differences in baseline CT usage and hospitalizations might explain the differences in the results compared to our study. During the study period, no national guidelines were available. Still, some previously published algorithms (for example, PECARN) may have affected clinical judgment, which might explain the number of patients who fulfilled the SCN16 criteria.

The strength of this study lies in the collection of data from real-life medical records, thereby enhancing the reliability of the findings. The diagnoses and injury severity levels were re-evaluated for each patient, and all available medical records data were screened. There are, nevertheless, several limitations. Because this study was conducted retrospectively, the amount of available information was limited. Especially during the first years included, there were substantial differences in the amount of information recorded in the medical records. It is possible that not all the symptoms were recorded. Other weaknesses include the lack of re-evaluation of neuroimaging examinations, particularly in patients with both head CT and MRI, as prior imaging may influence MRI interpretation. However, the findings are consistent with prior literature and are therefore considered reasonably reliable. In addition, we acknowledge that the study design does not allow assessment of the underutilization of neuroimaging, which should be regarded as a limitation. The study population, which consists of patients from a single paediatric tertiary care centre in Finland, is also a limitation. Therefore, the generalizability of the results is limited. The clinical decision-making, however, was based on the same information used in this study; thus, our study reflects the clinical reality of treating these patients.

## 5 Conclusions

Despite the increase in neuroimaging studies performed in the acute tertiary care setting at our centre between 2010 and 2016, patient selection for acute neuroimaging of paediatric head injuries was well aligned with the SCN16 guidelines published later. Using neuroimaging guidelines helps ensure appropriate patient selection for neuroimaging and hospital admissions. During 2010–2016, the number of neuroimaging examinations increased, while the number of traumatic intracranial findings remained stable or decreased, suggesting a change in neuroimaging practices at our centre at the beginning of our study period.

Based on the information gathered in this study and previous studies, the possibility of using MRI as an acute neuroimaging modality in the selected group of patients with head injury could be considered. Using MRI as the only neuroimaging modality for selected patients (if available) reduces the exposure to ionizing radiation and the need for repetitive neuroimaging. It is also important to note that advanced neuroimaging techniques, such as DTI, SWI, and possibly fMRI, are crucial in assessing functional outcomes following head injury.

Further research is needed to assess the selection of paediatric patients for MRI as the only neuromaging modality. Most guidelines are intended to support decision-making in acute settings, but less is known about neuroimaging indications and benefits of head injury patients after the acute phase.

## Data availability statement

Due to ethical restrictions, the data will only be available from the corresponding author upon reasonable request.

## Ethics approval

The study was conducted following protocols approved by Turku University Hospital. The Ethics Committee and informed consent requirements were waived by law, and participants were not contacted due to the retrospective registry study design.

## Contributors

Nina Erkinjuntti contributed to the study design, data collection, analysis, and interpretation; drafted the manuscript and implemented revisions based on co-authors' feedback.

Jussi P. Posti contributed to the study design, data collection (when a second opinion was needed), data interpretation, and article revision.

Tuire Lähdesmäki contributed to the study design, data collection (when a second opinion was needed), data interpretation, and article revision.

## Funding

This work was funded by the Arvo and Lea Ylppö Foundation [20250024], 10.13039/501100007417Paulo Foundation, and State Research Funding of the 10.13039/501100011797Turku University Hospital District. TL was supported by the Pä ivikki and Sakari Sohlberg Foundation and State Research Funding of the 10.13039/501100011797Turku University Hospital District. JPP is supported by the 10.13039/501100002341Research Council of Finland, the 10.13039/501100002341Academy of Finland, and the Sigrid Jusélius Foundation. The funders had no role in the study design, data collection and analysis, decision to publish, or preparation of the manuscript.

## Declaration of competing interest

The authors declare that they have no known competing financial interests or personal relationships that could have appeared to influence the work reported in this paper.
