# Supplementary material for: Evaluating clinical characteristics and neuroimaging indications of paediatric traumatic brain injury patients using Scandinavian paediatric traumatic brain injury guidelines in Southwest Finland
Source: Brain Spine. 2026 Feb 12;6:105969. doi: 10.1016/j.bas.2026.105969 (PMC12925293; doi:10.1016/j.bas.2026.105969)
Supplement: Multimedia component 1 [file mmc1.docx]

**Supplementary tables**

| **Supplementary Table 1** |  |  |  |  |  |
| --- | --- | --- | --- | --- | --- |
| **Causes of injury among age groups and sex (n=637)** | | | | | |
| **Cause of injury** | Incidental falls | Other non-intentional injury | Violence/assault | Traffic | Total |
| **Male** |  |  |  |  |  |
| <1 year of age | 1 (0.2) | 11 (1.7) | 2 (0.3) | 0 | 14 (2.2) |
| 1-5 years | 59 (9.3) | 15 (2.4) | 0 | 2 (0.3) | 76 (11.9) |
| 6-10 years | 54 (8.5) | 37 (5.8) | 2 (0.3) | 18 (2.8) | 111 (17.4) |
| 11-15 years | 34 (5.3) | 90 (14.1) | 9 (1.4) | 37 (5.8) | 170 (26.7) |
| **Female** |  |  |  |  |  |
| < 1 year of age | 0 | 14 (2.2) | 0 | 0 | 14 (2.2) |
| 1-5 years | 47 (7.4) | 13 (2.0) | 0 | 2 (0.3) | 62 (9.7) |
| 6-10 years | 33 (5.2) | 18 (2.8) | 1 (0.2) | 5 (0.8) | 56 (8.8) |
| 11-15 years | 62 (9.7) | 29 (4.6) | 3 (0.5) | 39 (6.1) | 134 (21.0) |
| **Both sexes** |  |  |  |  |  |
| < 1 year of age | 1 (0.2) | 25 (3.9) | 2 (0.3) | 0 | 28 (4.4) |
| 1-5 years | 106 (16.6) | 28 (4.4) | 0 | 4 (0.6) | 138 (21.7) |
| 6-10 years | 87 (13.7) | 55 (8.6) | 2 (0.3) | 23 (3.6) | 167 (26.2) |
| 11-15 years | 96 (15.1) | 119 (18.7) | 13 (2.0) | 76 (11.9) | 304 (47.7) |
| Data is the number of patients (% of the study population). | | |  |  |  |

| **Supplementary Table 2** |  |  |  |  |  |  |  |  |  |  |  |
| --- | --- | --- | --- | --- | --- | --- | --- | --- | --- | --- | --- |
| Distribution of extra-cerebral injuries among sex, age groups, and cause of injury in the whole study population (n=637) | | | | | | | | | | | |
| Extracerebral injuries | Sex | | Age | | | | Cause of injury | | | | All |
| Extracerebral injuries | Male | Female | < 1 year | 1-5 years | 6-10 years | 11-15 years | Incidental falls | Other non-intentional injuries | Violence/assault | Traffic | All |
| No. Injuries | 309 (56.7) | 236 (43.3) | 28 (5.1) | 116 (21.3) | 140 (25.7) | 261 (47.9) | 202 (37.1) | 184 (33.8) | 16 (2.9) | 143 (26.2) | 545 |
| Limb and pelvic fractures | 25 (67.6) | 12 (32.4) | 1 (2.7) | 5 (13.5) | 8 (21.6) | 23 (62.2) | 11 (29.7) | 4 (10.8) | 0 | 22 (59.5) | 37 (6.8) |
| Injuries of the trunk and thorax (incl. Internal organs) | 10 (35.7) | 18 (64.3) | 2 (7.1) | 6 (21.4) | 5 (17.9) | 15 (53.6) | 6 (21.4) | 5 (17.9) | 4 (14.3) | 13 (46.4) | 28 (5.1) |
| Superficial injuries | 251 (58.6) | 177 (41.4) | 25 (5.8) | 99 (23.1) | 115 (26.9) | 189 (44.2) | 168 (39.2) | 161 (37.6) | 11 (2.6) | 88 (20.6) | 428 (78.5) |
| Spinal injuries of any level and neck ligament injuries | 7 (35.0) | 13 (65.0) | 0 | 4 (2.0) | 3 (15.0) | 13 (65.0) | 7 (35.0) | 5 (25.0) | 1 (5.0) | 7 (35.0) | 20 (3.7) |
| Other injuries | 16 (50.0) | 16 (50.0) | 0 | 2 (6.3) | 9 (28.1) | 21 (65.6) | 10 (31.3) | 9 (28.1) | 0 | 13 (40.6) | 32 (5.9) |
| The data is the number of injuries (% of the injuries). | | | | | | | | | | | |

| **SupplementaryTable 3** |  |  |  |  |  |
| --- | --- | --- | --- | --- | --- |
| **Patient demographics, neuroimaging, neurosurgical interventions, hospital admissions and extra-cerebral injuries among patients classified according to SCN guidelines** | | | | | |
|  | **Mild- high risk** | **Mild- moderate risk** | **Mild-low risk** | **Minimal risk** | p |
| **No. Of patients** | 158 | 79 | 238 | 37 |  |
| **Sex** |  |  |  |  | *0.013* |
| Male | 130 (82.2) | 55 (69.6) | 125 (52.5) | 20 (54.1) |  |
| Female | 55 (34.8) | 24 (30.4) | 113 (47.5) | 17 (45.9) |  |
| **Age** |  |  |  |  | *<0.001* |
| < 1 year of age | 1 (0.6) | 1 (1.3) | 17 (7.1) | 0 |  |
| 1-5 years | 37 (23.4) | 12 (15.2) | 56 (23.5) | 4 (10.8) |  |
| 6-10 years | 41 (25.9) | 19 (24.1) | 70 (29.4) | 6 (16.2) |  |
| 11-15 years | 79 (50.0) | 47 (59.5) | 95 (39.9) | 27 (73.0) |  |
| **Cause of injury** |  |  |  |  | *<0.001* |
| Incidental falls | 89 (56.3) | 28 (35.4) | 104 (43.7) | 12 (32.4) |  |
| Other non-intentional injury | 53 (33.5) | 31 (39.2) | 82 (34.5) | 6 (16.2) |  |
| Violence/assault | 5 (3.2) | 2 (2.5) | 4 (1.7) | 1 (2.7) |  |
| Traffic | 11 (7.0) | 18 (22.8) | 48 (20.2) | 18 (48.6) |  |
| **CT imaging** | 95 (60.1) | 65 (82.3) | 165 (69.3) | 31 (83.8) | 0.198 |
| TIP on CT | 13 (8.2) | 8 (10.1) | 18 (7.6) | 0 |  |
| aCT imaging | 85 (53.8) | 56 (70.9) | 148 (62.2) | 28 (75.7) | 0.152 |
| TIP on aCT | 10 (6.3) | 8 (10.1) | 12 (5.0) | 0 |  |
| **MRI imaging** | 105 (66.5) | 43 (54.4) | 135 (56.7) | 9 (24.3) | 0.093 |
| TIP on MRI | 19 (12.0) | 14 (17.7) | 32 (13.4) | 0 |  |
| aMRI imaging | 28 (17.7) | 2 (2.5) | 31 (13.0) | 5 (13.5) | 0.390 |
| TIP on MRI | 3 (1.9) | 0 | 7 (2.9) | 0 |  |
| **Neurosurgical interventions** | 6 (3.8) | 1 (1.3) | 5 (2.1) | 0 | 0.422 |
| **Hospital admissions** | 111 (70.3) | 58 (73.4) | 174 (73.1) | 22 (59.5) | 0.366 |
| **Extra-cerebral injury** | 97 (61.4) | 58 (73.4) | 175 (73.5) | 22 (59.5) | *0.032* |
| Data is the number of patients, % of the group | | | | | |
| TIP= traumatic intracranial pathology, aCT=acute CT imaging of the head, aMRI=acute MRI imaging of the head | | | | | |

| **Supplementary Table 4** |  |  |  |  |  |
| --- | --- | --- | --- | --- | --- |
| Distribution of extra-cerebral injuries among SCN subgroups | |  |  |  |  |
|  | Minimal risk | Mild-low risk | Mild-moderate risk | Mild-high risk | All |
| No. patients (% of the population) | 37 (7.2) | 238 (46.5) | 79 (15.4) | 158 (30.9) | 512 |
| No. injuries (% of all injuries) | 25 (5.9) | 212 (49.8) | 80 (18.8) | 109 (25.5) | 426 |
| Limb and pelvic fractures | 2 (7.4) | 16 (59.2) | 5 (18.5) | 4 (14.9) | 27 (6.3) |
| Injuries of the trunk and thorax (incl. Internal organs) | 1 (6.0) | 7 (41.1) | 7 (41.1) | 2 (11.8) | 17 (4.0) |
| Superficial injuries | 18 (5.2) | 170 (49.4) | 63 (18.3) | 93 (27.1) | 344 (80.8) |
| Spinal injuries of any level and neck ligament injuries | 2 (15.4) | 7 (53.8) | 0 | 4 (30.8) | 13 (3.0) |
| Other injuries | 2 (8.0) | 12 (48.0) | 5 (20.0) | 6 (24.0) | 25 (5.9) |
| The data is % of injuries. |  |  |  |  |  |
